# Supplementary material for: Residential Environment Assessment by Older Adults in Nursing Homes during COVID-19 Outbreak
Source: Int J Environ Res Public Health. 2022 Dec 6;19(23):16354. doi: 10.3390/ijerph192316354 (PMC9739633; doi:10.3390/ijerph192316354)
Supplement: Supplementary file 1 [file ijerph-19-16354-s001.zip › ijerph-2021916-supplementary.pdf]

**Table S1.** Principal Components Analysis of the Assessment of the Residential Environment scale (EVAER)

| Items | Components | | | | Comunalities |
|---|---|---|---|---|---|
| | **1: Relationships (workers, residents, family, friends)** | **2: Nursing home aspects (management, security, amenities)** | **3: Mobility (outdoor and indoor)** | **4: Privacy space** | |
| 2. Relationships with nursing home workers | **0.80** | | | 0.34 | 0.81 |
| 1. Friendships with other residents | **0.75** | | | | 0.67 |
| 3. Relationships with family members (e.g., visits in person, by phone, email, other means) | **0.66** | | 0.35 | | 0.58 |
| 4. Relationships with friends (e.g., visits in person, by phone, email, other means) | **0.56** | | 0.56 | | 0.69 |
| 10. How well their residential home has managed during the COVID-19 pandemic | | **0.85** | | | 0.76 |
| 7. Their perception of safety in the residential home | 0.31 | **0.77** | | | 0.72 |
| 8. Characteristics and quality of the residential home's amenities; (e.g., room size and design, natural lighting, noise level, temperature, green areas) | | **0.66** | | 0.42 | 0.64 |
| 6. The chance to go outside the residential home for different activities (e.g., walking around the neighbourhood or town, shopping, doing other leisure activities, visiting friends) (outdoor accessibility) | | | **0.87** | | 0.80 |
| 5. Getting around easily within the residential home (e.g., without: obstacles or barriers, ramps, stairs, …) (indoor accessibility) | | | **0.76** | | 0.74 |
| 9. Having a space for privacy (having a single room or other personal space so as not to be disturbed) | | | | **0.86** | 0.83 |

Extraction method: Principal Component Analysis. Rotation method: Varimax with Kaiser normalization (rotation converged in 8 iterations).
Total variance explained: 72.2%
Factor loadings ≥ 0.300 in absolute value are shown. In grey, secondary factors loadings.
The Kaiser-Meyer-Olkin (KMO) of sampling adequacy = 0.823. The Bartlett's Test: $\chi^2$ = 976.520, df = 45, p < 0.001.
The Cronbach's Alpha coefficient: for all items = 0.84; component 1 = 0.77; component 2 = 0.72; component 3 = 0.74; component 4 = no Cronbach's Alpha for this factor based on a single variable.

**Table S2.** Grouping of subjects according to residential assessment

| Subscales of the Residential Environment Assessment | Final Clusters Centres | | |
|---|---|---|---|
| | **Cluster 1: medium-high rating of all residential aspects** | **Cluster 2: low rating linked to mobility** | **Cluster 3: low rating of all residential aspects** |
| Relationships (workers, residents, family, friends) | **0.35** | −0.22 | **−1.50** |
| Nursing home aspects (management, security, amenities) | **0.28** | 0.35 | **−1.85** |
| Mobility (outdoor and indoor accessibility) | **0.51** | **−1.43** | **−1.13** |
| Space for privacy (having a single room or other personal space so as not to be disturbed) | **0.30** | 0.13 | **−1.65** |
| Valid number of cases: 421 | 301 | 65 | 55 |
| % | 71.5 | 15.4 | 13.1 |

The 98.3% of original grouped cases were correctly classified through Discriminant Analysis.

**Table S3.** Residential assessment clusters according to personal and contextual conditions

| Topic | ANOVA Tests f Between-Subjects Effects (1) | | | Chi-Square Tests (2) | | | |
|---|---|---|---|---|---|---|---|
| | **Variable (N; Minimum; Maximum; Mean)** | **F** | **Sig** | **Variable** | **Value** | **df** | **Sig** |
| **Sociodemographic features** | Age in years (447; 61; 99; 83.80) | 4.858 | 0.008 | Gender | 11.433 | 2 | 0.003 |
| | Level of education (447; 1.00; 5.00; 2.99) | 0.380 | 0.684 | Marital status (married/living with partner; separated/divorced; single; widow/er) | 28.008 | 6 | 0.000 |
| | Total number of children (447; 0; 9; 1.56) | 4.729 | 0.009 | | | | |
| **Residential environment** | | | | Room availability (Single; Shared with spouse/partner/family member; Shared with another resident) | 15.508 | 4 | 0.004 |
| | | | | Where did you spend most of your time during the COVID-19 lockdown? (Always in my room; I made limited use of different areas; Unrestricted use of all spaces in the care home) | 22.301 | 4 | 0.000 |
| **Media means for relationships** | | | | Devices or mobile systems used to maintain relationships with your family or friends (Through my own devices; Through other people's or residential home's devices; I do not have access to those devices; Don't know, don't know that technology) | 15.844 | 4 | 0.003 |
| **Coronavirus status** | | | | Coronavirus status (I have not had the disease; I have had the disease, but I am still suffering after-effects; I have had the disease, but I do not have any after-effects) | 0.673 | 4 | 0.955 |
| | Fear of COVID 19 Infection FCV-19S (447; 7; 35; 18,4) | 3.291 | 0.038 | | | | |
| | Worried about the COVID-19 pandemic (443; 1.00; 4.00; 2.82) | 0.433 | 0.649 | | | | |
| | Number of protective measures to keep yourself safe from coronavirus (447; 0; 7; 3.96) | 5.994 | 0.003 | | | | |
| **Physical health** | Number of previous diagnosed pathologies (439; 1; 12; 6.06) | 0.464 | 0.629 | | | | |

| | | | | | | | |
|---|---|---|---|---|---|---|---|
| | Number of medications taken (439; 1; 8; 3.44) | 1.514 | 0.222 | | | | |
| | Self-assessment of health status, 3G (447; 1.00; 3.00; 2.45) | 3.780 | 0.024 | | | | |
| **Mental health** | The Abbreviated Geriatric Depression Scale-Yesavage (GDS-5) (447; 0; 5; 1.67) | 11.506 | 0.000 | | | | |
| **Affects** | The Positive and Negative Affect Schedule (PANAS) (447; -12; 15; 2.74) | 9.706 | 0.000 | | | | |
| | How often do you feel lonely? (447; 1.00; 4.00; 1.94) | 9.777 | 0.000 | | | | |
| **Resilience** | The Brief Resilient Coping Scale (BRCS) (447; 4; 20; 15.86) | 5.801 | 0.003 | | | | |
| **Leisure activities** | | | | 4 clusters of leisure activities: Use of mobile devices; Rewarding activities, volunteering; Religious practice; Inactivity | 41,515 | 6 | 0.000 |
| **Quality of Life and Wellbeing** | The Global Quality of Life (the FUMAT scale, 24 items) (447; 45; 95; 79.64) | 14.903 | 0.000 | | | | |
| | Satisfaction with life in comparative perspective (before and during the COVID-19 pandemic) (443; 1.00; 3.00; 1.60) | 5.019 | 0.007 | | | | |
| | Self-perception of stay in the nursing home in comparative perspective (before and during the COVID-19 pandemic) (429; 1.00; 3.00; 2.12) | 16.468 | 0.000 | | | | |
| **Ageing self-assessment** | Attitude Toward Own Aging subscale (ATOA) (408; 0; 5; 1.91) | 3.687 | 0.026 | | | | |

(1) ANOVA used with scale and ordinal variables

(2) Chi-Square test used with nominal variables

In grey shadow, non-significant variables and not used in subsequent analyses
